# Regulation of plasma lipid homeostasis by hepatic lipoprotein lipase in adult mice[S]

**DOI:** 10.1194/jlr.M065011

**Published:** 2016-07

**Authors:** Gan Liu, Jun-Nan Xu, Dong Liu, Qingli Ding, Meng-Na Liu, Rong Chen, Mengdi Fan, Ye Zhang, Chao Zheng, Da-Jin Zou, Jianxin Lyu, Weiping J. Zhang

**Affiliations:** Department of Pathophysiology* Second Military Medical University, Shanghai 200433, China; Obesity and Diabetes Research Center,† Second Military Medical University, Shanghai 200433, China; Key Laboratory of Laboratory Medicine,§ Ministry of Education of China, Wenchou Medical University School of Laboratory Medicine and Life Sciences, Wenchou, Zhejiang 325035, China; Department of Endocrinology,** Changhai Hospital, Shanghai 200433, China; Department of Endocrinology,†† Second Affiliated Hospital, Wenchou Medical University, Wenchou, Zhejiang 325000, China

**Keywords:** lipid metabolism, liver, hypertriglyceridemia, knockout mice

## Abstract

LPL is a pivotal rate-limiting enzyme to catalyze the hydrolysis of TG in circulation, and plays a critical role in regulating lipid metabolism. However, little attention has been paid to LPL in the adult liver due to its relatively low expression. Here we show that endogenous hepatic LPL plays an important physiological role in plasma lipid homeostasis in adult mice. We generated a mouse model with the *Lpl* gene specifically ablated in hepatocytes with the Cre/LoxP approach, and found that specific deletion of hepatic *Lpl* resulted in a significant decrease in plasma LPL contents and activity. As a result, the postprandial TG clearance was markedly impaired, and plasma TG and cholesterol levels were significantly elevated. However, deficiency of hepatic *Lpl* did not change the liver TG and cholesterol contents or glucose homeostasis. Taken together, our study reveals that hepatic LPL is involved in the regulation of plasma LPL activity and lipid homeostasis.

LPL is a multifunctional protein and plays a major role in the metabolism and transport of lipids (1). As a rate-limiting enzyme, it catalyzes the hydrolysis of core TG in circulating chylomicrons and VLDLs. As a result, the released FFAs and monoacylglycerol are, in part, taken up by local tissues for energy storage or utilization (2). LPL is principally synthesized and secreted by the parenchymal cells of tissues including heart, adipose, skeletal muscle, and brain, as well as macrophages, and then translocated to the luminal surface of vascular endothelial cells, where lipolytic processing occurs (3). Whole-body deficiency of *Lpl* results in severe hypertriglyceridemia and neonatal death in mice (4). Tissue-specific overexpression or deletion of LPL in mouse models suggests that LPL controls entry of fatty acids into the tissues, such as skeletal muscle, adipose, heart, and neurons (5–7). Moreover, manipulation of *Lpl* expression in a given tissue causes imbalances in the partitioning of fatty acids among peripheral tissues, which consequently have major effects on glucose and lipid metabolism. For example, the mice with specific overexpression of LPL in skeletal muscle are insulin resistant, with a marked increase in muscle TG content and blood glucose levels (5); the mice lacking the *Lpl* gene specifically in neurons are hyperphagic and obese, with elevations in the hypothalamic orexigenic neuropeptides, such as AgRP and NPY (7). Thus LPL functions as a key regulator of lipid metabolism and as a gatekeeper of energy partitioning.

However, the biological significance of LPL in adult liver is not well appreciated due to its relatively low expression level (8). LPL expression is developmentally regulated in the liver, with a distinct pattern relative to extrahepatic tissues. In rodents, LPL activity rapidly declines during the first few weeks after birth. As a result, in adult liver, LPL expression was reported to be only detected in some scattered cells by immunohistochemistry, which were most likely Kupffer cells (8). Therefore, liver LPL is putatively regarded to be mainly expressed at embryonic and postnatal stages, when it shunts circulating TG to the liver to provide more energy for the production of VLDLs and ketone bodies at times of metabolic stress (9). Of note, LPL expression is positively regulated by the nuclear receptor, LXR, in the liver (10). Moreover, liver-specific overexpression of LPL leads to a 2-fold increase in liver TG content and insulin resistance in mice (5). This raises the interesting subject that liver LPL may have a physiologically or pathophysiologically significant role in lipid metabolism at adulthood. To test this hypothesis, we generated hepatocyte-specific *Lpl* knockout mice. Our findings suggest that liver LPL is physiologically active at adulthood and critically involved in the regulation of lipid metabolism.

## MATERIALS AND METHODS

### Animals

Liver-specific *Lpl* knockout mice were generated by crossing *Lpl*^flox/flox^ (Jackson Laboratory, #6503) mice (6) with albumin-Cre transgenic mice (11, 12). The mice were genotyped by PCR analysis of tail genomic DNA with primers producing a 515 bp band for the floxed allele and a 465 bp band for the WT band (6). To delete the *Lpl* gene in adult liver, *Lpl*^flox/flox^ mice were ip injected with recombinant adenoviruses expressing Cre (Ad-Cre) or GFP (Ad-GFP) as control (0.2 optical density per mouse), and maintained on chow that was fed for another 2 weeks before experiments. The recombinant adenoviruses were purified by CsCl ultracentrifugation, and subjected to dialysis against PBS before titration in 293A cells (13). The primers for Cre PCR amplification were described previously (12). All mice were maintained on an autoclaved chow diet in filter-topped cages in a specific pathogen free animal room. In all animal experiments, littermates carrying the loxP-flanked alleles but lacking Cre recombinase were used as WT controls. For diet-induced obesity, mice were fed a high-fat diet (HFD) (20.1% carbohydrate, 59.9% fat, 20.0% protein) for 16 weeks at 4 weeks of age. All animal experiments were done following institutional guidelines.

### Plasma metabolite measurements

Blood was collected from the retro-orbital plexus into heparinized tubes after a 6 h fast. The tubes were placed on ice, centrifuged at 4°C, and stored at −80°C. Plasma and tissue TG concentrations were determined using the serum TG determination kit, TR0100 (Sigma, St. Louis, MO). Plasma FFAs were determined using a kit (catalog #294-63601) from WAKO Chemicals (Sopachem, Ochten, The Netherlands). For lipoprotein separation, plasma samples pooled from five mice per genotype were resolved by fast-performance LC on a gel filtration column, Superose 6 (Bio-Rad), with the eluates collected in 0.5 ml fractions at a flow rate of 0.5 ml/min, and TG levels were measured with a TG quantification colorimetric/fluorometric kit (Biovision).

### Liver TG secretion and postprandial TG test

Liver TG was extracted with acetone for colorimetric assays. The liver TG production test was performed by ip injection of poloxamer 407 (1 mg/g; Sigma) into 16 h-fasted mice, and plasma TG was measured at 0, 1, 2, 6, and 24 h after injection (14). The postprandial TG test was performed on 16 h-fasted mice by gavage of corn oil (10 μl/g; Sigma), and plasma TG was measured at 0, 2, 4, 6, and 8 h after gavage.

### Glucose and insulin tolerance test

Glucose and insulin tolerances tests were performed as described previously (15). Following the overnight fast (6 h), glucose and insulin tolerance tests were by bolus ip injection of glucose (2 g/kg; Sigma) or insulin (0.51 U/kg; Sigma), respectively. Blood glucose was measured using a glucose glucometer by tail bleeding at 0, 15, 30, 60, and 120 min after injection.

### Quantitative RT-PCR

Total RNA was isolated from liver samples by Trizol reagent (Invitrogen, Carlsbad, CA) and cDNA was synthesized by using reverse transcription reagent (Promega, Madison, WI). Quantitative reverse transcription PCR (qRT-PCR) was performed using a standard SYBR green PCR kit (Promega, Madison, WI), and PCR-specific amplification was conducted in an Eppendorf real-time PCR machine (12). The expression of genes was calculated with the 2^−(ΔΔCt)^ method. The primer sequence for *Lpl* was: forward, CAGAGTTTGACCGCCTTCC; reverse, AATTTGCTTTCGATGTCTGAGAA.

### LPL protein and activity assays

For postheparin plasma LPL levels, blood was collected at 10 min after tail vein injection of heparin (0.1 U/g body weight) diluted in PBS. Plasma LPL levels were measured using specific ELISA kits (catalog #SEA386Mu; Cloud-Clone Corp., Houston, TX) following the manufacturer’s instructions. For Western blot analysis, 15 μl of plasma was loaded on SDS-PAGE gel to resolve before probing with anti-LPL antibody (Santa Cruz Biotechnology). Plasma LPL activity was determined with ^3^H-triolein as substrate tracer (16). Briefly, 10 μl samples were mixed with the anhydrous emulsion containing 0.01% ^3^H-triolein in the presence of heparin and heat-inactivated fast rat serum. After reaction at 37°C for 60 min, the mixture was extracted with an organic solvent (21% methanol, 44% chloroform, 35% heptane), and 1 ml of the upper aqueous phase was removed to mix with 4 ml of scintillator liquid for counting in a spectrometer (Beckman LS 6500). To abolish LPL activity, samples were pretreated with 1 M NaCl, and LPL activities were obtained by subtracting NaCl-treated enzyme activities from total enzyme activities.

### Statistics

Data are presented as mean ± SEM. Statistical significance between two experimental groups was assessed using a Student’s *t*-test. Significance was accepted at the level of *P* < 0.05 (*).

## RESULTS

### Expression of LPL in adult liver

**Fig. 1.**
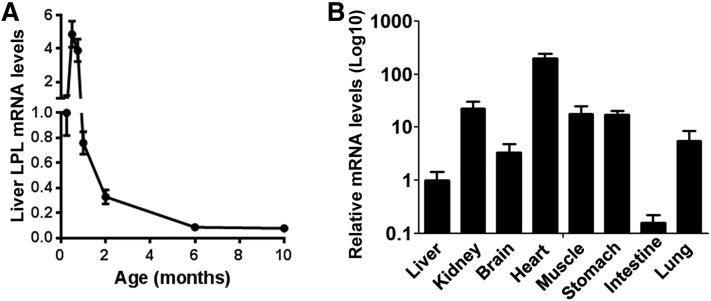
Characterization of liver *Lpl* mRNA expression in adult mice. *Lpl* mRNA expression was detected by real-time RT-PCR in the tissues from normal C57BL/6 mice. A: *Lpl* mRNA expression in the liver at the indicated ages. B: *Lpl* mRNA expression in the indicated tissues from adult mice at the age of ∼3–4 months. *Lpl* mRNA levels were normalized by internal control, 36B4 (n = ∼4–6).

To characterize the expression pattern of LPL in postnatal liver, we first performed quantitative RT-PCR analysis. *Lpl* mRNA levels were maintained at relatively high levels in the liver during the first 3 weeks after birth, and declined rapidly afterwards (**Fig. 1A**). Interestingly, *Lpl* mRNA levels still showed a steady tendency of downregulation in adult liver between 2 and 6 months of age. As a result, *Lpl* mRNA was expressed at quite low levels in adult liver compared with heart (∼200-fold lower), skeletal muscle (∼20-fold lower), kidney, lung, and brain (∼4-fold lower). Nevertheless, liver *Lpl* mRNA levels were still significantly higher than those in the small intestine (∼6-fold higher) (Fig. 1B).

### Generation of hepatocyte-specific *Lpl* knockout mice

**Fig. 2.**
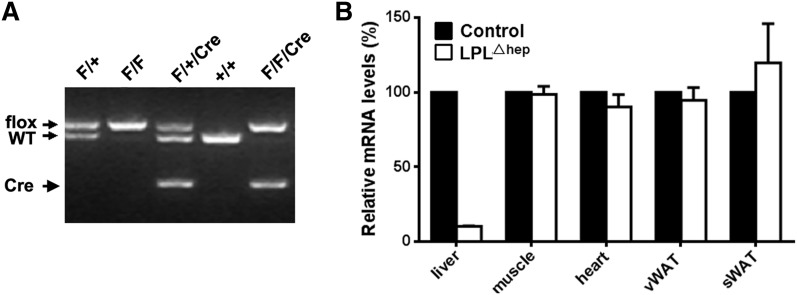
Generation of hepatocyte-specific *Lpl* knockout mice. A: PCR analysis of tail genomic DNA distinguishing the *Lpl*^flox^ allele and WT allele. PCR products in the size of 515 bp and 465 bp correspond to the floxed and WT alleles, respectively. Genotypes are indicated above each lane. F, floxed allele. B: The adult LPL^Δhep^ mice displayed a substantial and tissue-specific reduction in *Lpl* mRNA levels in the liver (n = ∼4–6 per group). sWAT, subcutaneous white adipose tissue; vWAT, visceral white adipose tissue.

To evaluate the potential role of LPL in adult liver, we generated hepatocyte-specific *Lpl* knockout mice (hereafter LPL^Δhep^) by crossing *Lpl*^flox/flox^ mice with the Alb-Cre driver line, which expresses the Cre recombinase under the albumin promoter (11). Genotyping was performed by PCR analysis of tail genomic DNA, which distinguished the floxed *Lpl* allele from WT (**Fig. 2A**). RT-PCR analysis showed that liver *Lpl* mRNA levels were reduced by 90% in adult mutant mice compared with the control group (Fig. 2B), suggesting that the hepatocytes, rather than endothelial or resident Kupffer cells, are the main cellular sources of liver LPL in adult WT mice. However, liver LPL protein was not detected by immunoblot analysis even in adult control mice, which was most likely due to its low expression levels. Importantly, hepatocyte-specific deletion of *Lpl* did not significantly alter *Lpl* mRNA levels in other peripheral tissues, such as heart, skeletal muscle, and white adipose. These results indicated that liver *Lpl* was efficiently and specifically deleted in *Lpl*^Δhep^ mice.

### Deletion of liver LPL decreases plasma LPL activity

**Fig. 3.**
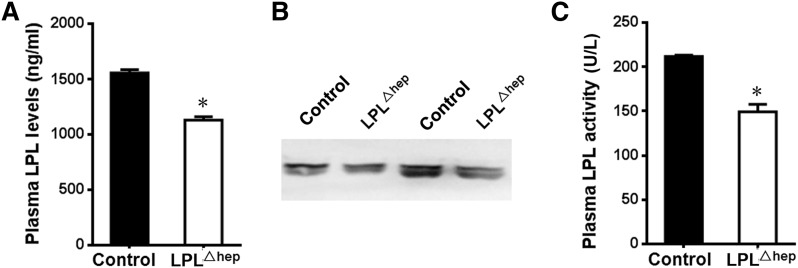
Deletion of the hepatic *Lpl* gene decreases plasma LPL contents and activity. The control and mutant adult mice were pretreated with heparin, and then their plasma was harvested for measuring LPL contents and activity. A, B: Plasma LPL contents were determined by ELISA (A) and Western blot (B). Plasma (15 μl) from paired mice was resolved by SDS-PAGE gel before probing with anti-LPL antibody. C: Plasma LPL activity was decreased by the deletion of hepatic *Lpl*. Results represent mean ± SEM. *P < 0.05 versus control; n = 4–5 for each group.

LPL is rapidly transported and anchored onto the luminal surface of vascular endothelial cells after secretion, which can be released into circulation by heparin (17–19). To determine whether deletion of liver *Lpl* affects plasma LPL activity, we first measured plasma LPL levels in heparinized mice by ELISA. Surprisingly, *Lpl*^Δhep^ mice displayed a 28.7% decrease in plasma LPL levels compared with control mice (**Fig. 3A**). In addition, Western blot also showed a significant decrease of LPL protein in the plasma from mutant mice (Fig. 3B). Consistently, LPL activity measures revealed that plasma LPL activity was reduced by 27.2% in the mutant mice (Fig. 3C). Furthermore, deletion of liver LPL did not lead to significant changes of mRNA expression levels of *Lipc* in the liver (supplementary Fig. 1), which encodes hepatic lipase and plays a critical role in lipid homeostasis, mainly by hydrolyzing TG in plasma lipoprotein (20). These data suggested that liver plays an important role in the regulation of plasma LPL mass and activity under normal physiological conditions, albeit its quite low expression.

### Disturbed lipid metabolism in the absence of liver LPL

**Fig. 4.**
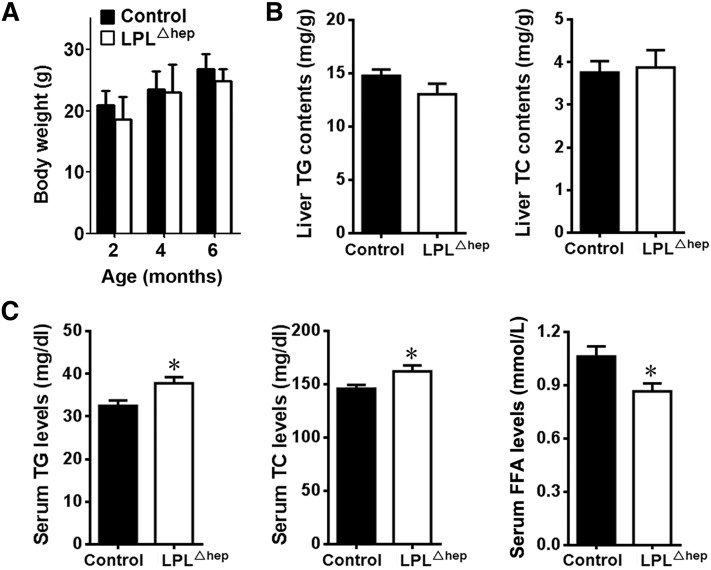
Disruption of liver LPL altered plasma lipid metabolism. The control and *Lpl*^Δhep^ male mice at the age of ∼2–6 months were fed a normal chow diet. A: Body weight was not affected at the age of ∼2–6 months. B: The TG and TC contents in the liver were not affected by the deletion of hepatic *Lpl*. C: LPL^Δhep^ mice showed an increase in fasting plasma TG and TC levels and a decrease in plasma FFA levels. The mice were fasted for 6 h. Results represent the mean ± SEM. *P < 0.05 versus control; n = 6–10 for each group.

Mice with homozygous deletion of *Lpl* in liver survived into adulthood without gross abnormalities on the normal chow diet. Both male and female adult LPL^Δhep^ mice grew similarly as the sex-matched littermate controls (**Fig. 4A**). The liver from adult *Lpl*^Δhep^ mice was normal in appearance and size, and had similar contents of TG and total cholesterol (TC) as the control group (Fig. 4B). Interestingly, both male and female *Lpl*^Δhep^ mice exhibited a mild, but significant, increase in serum TG and TC levels compared with their control counterparts under the fasting condition (Fig. 4C, supplementary Fig. 2), whereas their serum FFA levels were significantly decreased. Fast-performance LC analysis demonstrated that TG contents were significantly increased in VLDL fractions of plasma lipoprotein from mutant mice compared with control mice (supplementary Fig. 3). These results suggested that liver LPL is involved in the regulation of plasma lipid metabolism in adult mice.

### Impaired TG clearance in the absence of liver LPL

**Fig. 5.**
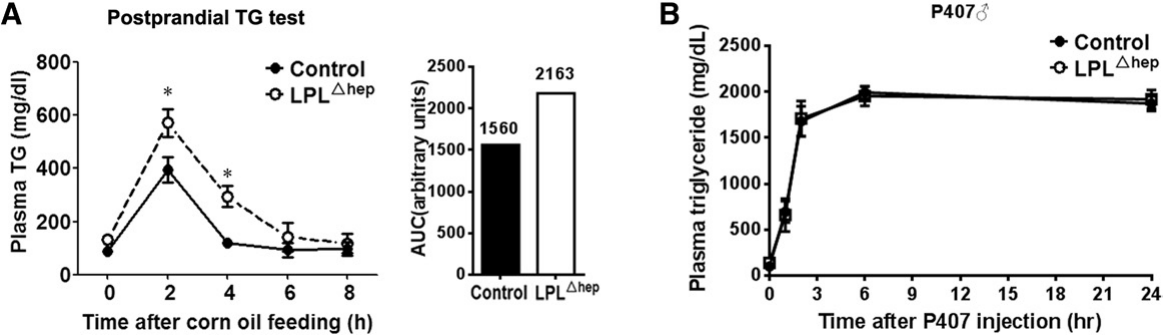
Disruption of liver LPL impaired postprandial TG clearance. A: Postprandial TG clearance test. Four-month-old male mice were administered corn oil by gavage following a 16 h fast, and plasma TG levels were determined at the indicated time points. Area under curve (AUC) was plotted with the indicated values. B: VLDL secretion test. Male mice at the age of ∼3–4 months were ip injected with the LPL inhibitor, P407, and plasma TG levels were determined at the indicated time points. Results represent the mean ± SEM. *P < 0.05 versus control; n = ∼4–5 for each group.

To understand the mechanisms by which liver LPL regulates plasma lipid homeostasis, we first assessed plasma TG clearance by postprandial TG test. Compared with the control group, LPL^Δhep^ mice displayed a substantial increase in plasma TG levels at 2 and 4 h after the gavage of corn oil, suggesting that TG clearance was greatly impaired in the absence of liver LPL (**Fig. 5A**). To determine whether hepatic TG secretion was altered in the absence of liver LPL, we further conducted a P407 test in which hepatic TG secretion is largely reflected by plasma TG accumulation due to the blockage of TG hydrolysis by the LPL inhibitor, P407. LPL^Δhep^ mice showed a similar response of plasma TG accumulation as control mice after P407 injection (Fig. 5B), indicative of their normal hepatic TG secretion. Taken together, these data suggested that liver LPL regulates plasma TG metabolism mainly through modulating TG clearance of plasma lipoproteins.

**Fig. 6.**
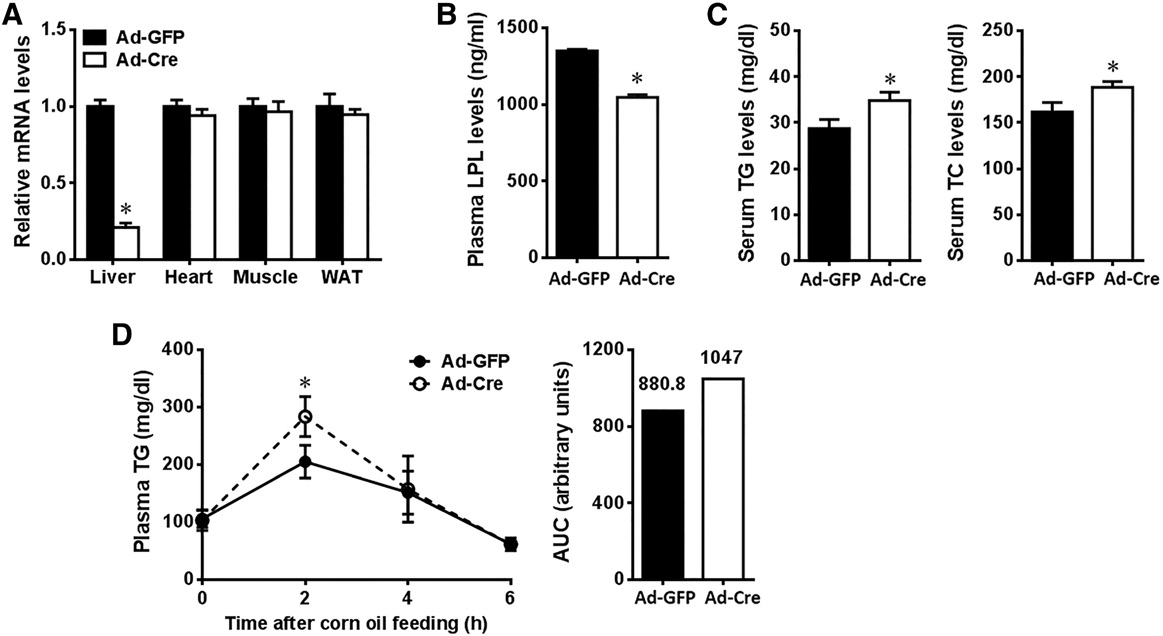
Effects of adenovirus-mediated deletion of liver LPL on plasma lipid metabolism in adult mice. Adult *Lpl*^flox/flox^ mice (∼2–3 months of age, male) were iv injected with Ad-Cre or Ad-GFP, and fed normal chow for 2 weeks before euthanization. A: *Lpl* mRNA levels in the liver and other tissues as indicated. B: Plasma LPL contents measured in heparinized mice by ELISA. C: Plasma lipid levels. D: Postprandial TG clearance test. Results represent the mean ± SEM. *P < 0.05 versus Ad-GFP control; n = ∼4–6 for each group. WAT, white adipose tissue; AUC, area under the curve.

Considering the possibility that Alb-Cre-mediated deletion of LPL in neonatal mice might affect liver development or the expression of LPL-related genes, which could influence TG and cholesterol metabolism, we took another approach to conditional ablation of the hepatic *Lpl* gene in adult mice. Cre recombinase was specifically delivered to the hepatocytes of adult *Lpl*^flox/flox^ mice by infusion of Cre-expressing recombinant adenoviruses (Ad-Cre). Two weeks after adenovirus delivery, the mice were subjected to phenotypic analysis. Compared with Ad-GFP control mice, Ad-Cre administration in *Lpl*^flox/flox^ mice reduced *Lpl* mRNA levels by 80.7% in liver, without significant changes of expression in other tissues, e.g., heart, skeletal muscle, and white adipose tissue (WAT) (**Fig. 6A**). As a result, plasma LPL mass was decreased by 20.2% in the heparinized mice lacking *Lpl* in adult liver (Fig. 6B), with significant elevation in plasma TG and TC levels (Fig. 6C), and TG clearance was significantly impaired at 2 h after corn oil gavage compared with control (Fig. 6D). These results suggest that hepatic LPL has a role in plasma lipid homeostasis at adulthood.

### Liver LPL ameliorates hyperlipidemia in HFD-induced obese mice

**Fig. 7.**
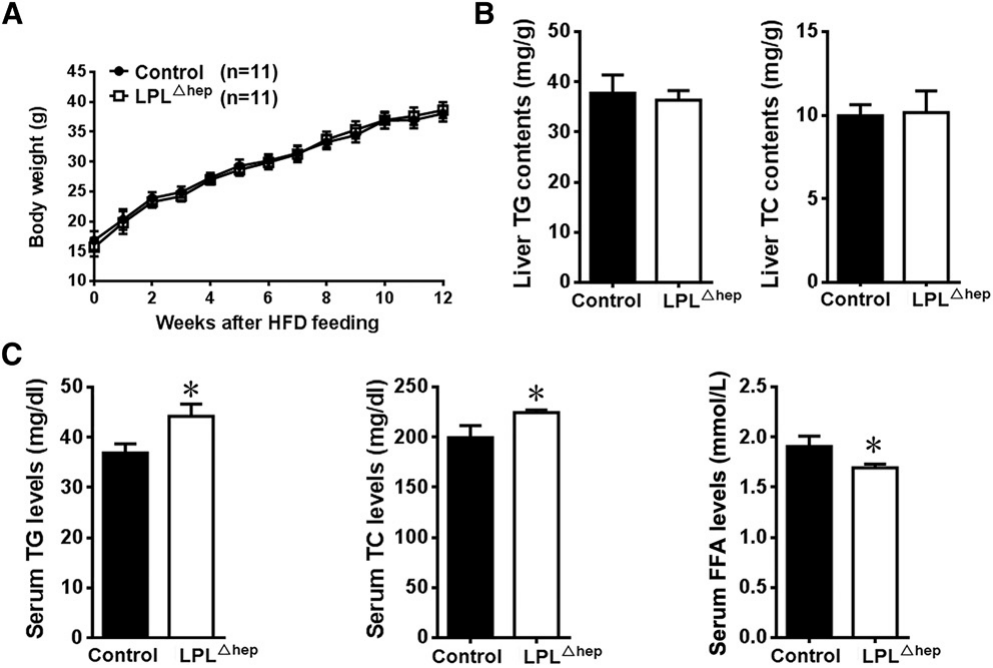
Liver LPL regulated plasma lipid metabolism in HFD-induced obese mice. Male control and *Lpl*^Δhep^ mice were fed a HFD for 3 months starting from 3 weeks of age. A: Body weight was not affected in *Lpl*^Δhep^ mice after ∼2–6 months of HFD feeding. B: The TG and TC contents in the liver were not affected in obese mice by the deletion of hepatic *Lpl*. C: Fasting plasma lipid levels after 3 months of HFD feeding. Results represent the mean ± SEM. *P < 0.05 versus control; n = ∼6–8 for each group.

To further determine the potential role of liver LPL in obesity, we fed male *Lpl*^Δhep^ and control mice with a HFD for 16 weeks. *Lpl*^Δhep^ and control mice showed comparable body weight gain (**Fig. 7A**). Compared with the chow diet, HFD feeding substantially increased the liver contents of TG and TC, however, neither TG nor TC was significantly different between control and mutant groups (Fig. 7B). Of note, HFD-fed mutant mice displayed a mild and significant elevation in fasting plasma TG and TC levels compared with the control group, whereas their plasma FFA levels showed a tendency of decrease without reaching statistical significance (Fig. 7C). Together, these data suggested that liver LPL facilitates the amelioration of hyperlipidemia in HFD-induced obese mice with no significant effects on tissue adiposity.

### Liver LPL does not contribute to glucose homeostasis

To explore whether liver LPL contributes to glucose homeostasis, we characterized glucose metabolism in the mutant mice. When fed the normal chow diet, both male and female LPL^Δhep^ mice did not display significant differences in blood glucose levels, fasting serum insulin levels, or homeostasis model assessment of insulin resistance compared with controls (supplementary Fig. 4A, B). In addition, glucose and insulin tolerance tests revealed that LPL^Δhep^ mice had normal glucose metabolism (supplementary Fig. 4C). When fed a HFD, both control and LPL^Δhep^ mice had comparable blood glucose levels (supplementary Fig. 4A) and exhibited severe glucose intolerance and insulin resistance, with no significant difference between the two genotypes (supplementary Fig. 4C). These results suggest that liver Lpl is dispensable for glucose homeostasis under physiological conditions and HFD-induced obesity.

## DISCUSSION

LPL functions as a key regulator for lipid metabolism and energy partitioning. In the past decades, most of the interest in LPL was focused on the extrahepatic tissues, such as heart, muscle, adipose tissue, and brain, as well as macrophages (1, 7). In contrast, little attention has been paid to LPL in adult liver due to its relatively low expression. In the present study, we, for the first time, provide compelling evidence that liver LPL is critically involved in the regulation of lipid metabolism at adulthood.

Our findings establish that liver significantly contributes to plasma LPL contents and activity in adult mice. Consistent with the previous reports, we show that LPL mRNA levels in adult liver are much lower than in heart, adipose tissues, and skeletal muscle. Furthermore, specific deletion of *Lpl* in hepatocytes leads to about a 90% reduction in *Lpl* mRNA levels in liver, which strongly supports that liver LPL is mainly derived from hepatocytes rather than Kupffer cells or vascular endothelial cells under physiological conditions. Unexpectedly, deletion of liver LPL results in an ∼27–29% decrease in heparin-released plasma LPL levels and activity. Obviously, this remarkable contribution of liver to plasma LPL levels is not proportional to its low expression levels of LPL, even if the tissue mass is considered. The discrepancy could be explained by the possibility that not all LPL protein expressed by parenchymal cells is accessible and released by heparin. Typically, after secretion, LPL is rapidly transported by GPIHBP1 and anchored onto the luminal surface of vascular endothelial cells, and LPL can be released into the circulation by heparin (17–19, 21). It is noteworthy that it was reported that the majority of LPL protein in heart was detected in myocytes (78%) rather than in capillary endothelium (22). Although the intracellular and extracellular distribution pattern of LPL has not been determined in other tissues, such as skeletal muscle, adipose tissue, or liver, this raised the possibility that cellular localization of LPL may be a highly regulated event and could be tissue dependent. In addition, the release efficiency of LPL into circulation by heparin could vary in different tissues, which may be regulated by local microenvironment, such as blood flow, nutrients, metabolites, etc. This possibility is partly supported by the previous report that LPL binding to vascular endothelium in the heart is regulated by nutritional status (19). Another possibility is that the LPL turnover in liver may be quite high. The number of LPL receptors on liver endothelial cells may be low, thereby favoring LPL secretion in the absence of heparin. A high concentration of LPL in the environment of liver cells would enhance LPL binding to lipoproteins and uptake of lipoproteins by liver receptors. Alternatively, it is also possible that liver LPL may regulate the LPL localization pattern in extrahepatic tissues through unidentified mechanisms. The exact mechanism by which liver LPL contributes to plasma LPL contents and activity needs further investigation.

Our findings also establish that liver LPL is involved in the regulation of plasma lipid homeostasis at adulthood, which is consistent with its contribution to plasma LPL. Deletion of liver *Lpl* results in a remarkable elevation in plasma TG levels and reduction in plasma FFA, which is largely due to impaired TG clearance of lipoproteins. In addition, plasma cholesterol levels were also increased in the mutant mice either on normal chow or HFD, which could be secondary to plasma TG metabolism. Considering the fact that liver TG or TC contents were not significantly affected by the loss of liver Lpl, we reason that the FFAs produced by liver LPL-mediated TG hydrolysis are most likely taken up by the extrahepatic tissues. This needs to be examined in the future.

Our study indicates that liver LPL has no significant role in glucose metabolism. It was previously reported that liver-specific transgenic overexpression of *Lpl* increases liver TG content and is associated with insulin resistance (5). Our results indicate that liver LPL has no physiological or pathophysiological effects on liver TG content and insulin resistance. This discrepancy implies that the effects of liver LPL may be dose dependent. Taken together, this study establishes a critical role of liver LPL in plasma TG metabolism, providing a better understanding of the cellular and molecular mechanisms of plasma lipid homeostasis.

## Supplementary Material

Supplemental Data

## References

[1] Wang H., and Eckel R. H. 2009 Lipoprotein lipase: from gene to obesity. Am. J. Physiol. Endocrinol. Metab. 297: E271–E288. 19318514 10.1152/ajpendo.90920.2008

[2] Hassing H. C., Surendran R. P., Mooij H. L., Stroes E. S., Nieuwdorp M., and Dallinga-Thie G. M. 2012 Pathophysiology of hypertriglyceridemia. Biochim. Biophys. Acta. 1821: 826–832. 22179026 10.1016/j.bbalip.2011.11.010

[3] Eisenberg S., Sehayek E., Olivecrona T., and Vlodavsky I. 1992 Lipoprotein lipase enhances binding of lipoproteins to heparan sulfate on cell surfaces and extracellular matrix. J. Clin. Invest. 90: 2013–2021. 1430223 10.1172/JCI116081 PMC443265

[4] Weinstock P. H., Bisgaier C. L., Aalto-Setala K., Radner H., Ramakrishnan R., Levak-Frank S., Essenburg A. D., Zechner R., and Breslow J. L. 1995 Severe hypertriglyceridemia, reduced high density lipoprotein, and neonatal death in lipoprotein lipase knockout mice. Mild hypertriglyceridemia with impaired very low density lipoprotein clearance in heterozygotes. J. Clin. Invest. 96: 2555–2568. 8675619 10.1172/JCI118319 PMC185959

[5] Kim J. K., Fillmore J. J., Chen Y., Yu C., Moore I. K., Pypaert M., Lutz E. P., Kako Y., Velez-Carrasco W., Goldberg I. J., 2001 Tissue-specific overexpression of lipoprotein lipase causes tissue-specific insulin resistance. Proc. Natl. Acad. Sci. USA. 98: 7522–7527. 11390966 10.1073/pnas.121164498 PMC34701

[6] Augustus A., Yagyu H., Haemmerle G., Bensadoun A., Vikramadithyan R. K., Park S. Y., Kim J. K., Zechner R., and Goldberg I. J. 2004 Cardiac-specific knock-out of lipoprotein lipase alters plasma lipoprotein triglyceride metabolism and cardiac gene expression. J. Biol. Chem. 279: 25050–25057. 15028738 10.1074/jbc.M401028200

[7] Wang H., Astarita G., Taussig M. D., Bharadwaj K. G., DiPatrizio N. V., Nave K. A., Piomelli D., Goldberg I. J., and Eckel R. H. 2011 Deficiency of lipoprotein lipase in neurons modifies the regulation of energy balance and leads to obesity. Cell Metab. 13: 105–113. 21195353 10.1016/j.cmet.2010.12.006 PMC3034302

[8] Camps L., Reina M., Llobera M., Bengtsson-Olivecrona G., Olivecrona T., and Vilaro S. 1991 Lipoprotein lipase in lungs, spleen, and liver: synthesis and distribution. J. Lipid Res. 32: 1877–1888. 1816319

[9] Merkel M., Weinstock P. H., Chajek-Shaul T., Radner H., Yin B., Breslow J. L., and Goldberg I. J. 1998 Lipoprotein lipase expression exclusively in liver. A mouse model for metabolism in the neonatal period and during cachexia. J. Clin. Invest. 102: 893–901. 9727057 10.1172/JCI2912 PMC508954

[10] Zhang Y., Repa J. J., Gauthier K., and Mangelsdorf D. J. 2001 Regulation of lipoprotein lipase by the oxysterol receptors, LXRalpha and LXRbeta. J. Biol. Chem. 276: 43018–43024. 11562371 10.1074/jbc.M107823200

[11] Postic C., Shiota M., Niswender K. D., Jetton T. L., Chen Y., Moates J. M., Shelton K. D., Lindner J., Cherrington A. D., and Magnuson M. A. 1999 Dual roles for glucokinase in glucose homeostasis as determined by liver and pancreatic beta cell-specific gene knock-outs using Cre recombinase. J. Biol. Chem. 274: 305–315. 9867845 10.1074/jbc.274.1.305

[12] Xie Z., Zhang H., Tsai W., Zhang Y., Du Y., Zhong J., Szpirer C., Zhu M., Cao X., Barton M. C., 2008 Zinc finger protein ZBTB20 is a key repressor of alpha-fetoprotein gene transcription in liver. Proc. Natl. Acad. Sci. USA. 105: 10859–10864. 18669658 10.1073/pnas.0800647105 PMC2504784

[13] Cao D., Ma X., Cai J., Luan J., Liu A. J., Yang R., Cao Y., Zhu X., Zhang H., Chen Y. X., 2016 ZBTB20 is required for anterior pituitary development and lactotrope specification. Nat. Commun. 7: 11121. 27079169 10.1038/ncomms11121 PMC4835541

[14] Millar J. S., Cromley D. A., McCoy M. G., Rader D. J., and Billheimer J. T. 2005 Determining hepatic triglyceride production in mice: comparison of poloxamer 407 with Triton WR-1339. J. Lipid Res. 46: 2023–2028. 15995182 10.1194/jlr.D500019-JLR200

[15] Zhang Y., Xie Z., Zhou L., Li L., Zhang H., Zhou G., Ma X., Herrera P. L., Liu Z., Grusby M. J., 2012 The zinc finger protein ZBTB20 regulates transcription of fructose-1,6-bisphosphatase 1 and beta cell function in mice. Gastroenterology. 142: 1571–1580. 22374165 10.1053/j.gastro.2012.02.043

[16] Hocquette J. F., Graulet B., and Olivecrona T. 1998 Lipoprotein lipase activity and mRNA levels in bovine tissues. Comp. Biochem. Physiol. B Biochem. Mol. Biol. 121: 201–212. 9972295 10.1016/s0305-0491(98)10090-1

[17] Korn E. D. 1955 Clearing factor, a heparin-activated lipoprotein lipase. II. Substrate specificity and activation of coconut oil. J. Biol. Chem. 215: 15–26. 14392138

[18] Babirak S. P., Iverius P. H., Fujimoto W. Y., and Brunzell J. D. 1989 Detection and characterization of the heterozygote state for lipoprotein lipase deficiency. Arteriosclerosis. 9: 326–334. 2719595 10.1161/01.atv.9.3.326

[19] Ruge T., Bergo M., Hultin M., Olivecrona G., and Olivecrona T. 2000 Nutritional regulation of binding sites for lipoprotein lipase in rat heart. Am. J. Physiol. Endocrinol. Metab. 278: E211–E218. 10662704 10.1152/ajpendo.2000.278.2.E211

[20] Santamarina-Fojo S., Gonzalez-Navarro H., Freeman L., Wagner E., and Nong Z. 2004 Hepatic lipase, lipoprotein metabolism, and atherogenesis. Arterioscler. Thromb. Vasc. Biol. 24: 1750–1754. 15284087 10.1161/01.ATV.0000140818.00570.2d

[21] Davies B. S., Beigneux A. P., Barnes R. H. II, Tu Y., Gin P., Weinstein M. M., Nobumori C., Nyren R., Goldberg I., Olivecrona G., 2010 GPIHBP1 is responsible for the entry of lipoprotein lipase into capillaries. Cell Metab. 12: 42–52. 20620994 10.1016/j.cmet.2010.04.016 PMC2913606

[22] Blanchette-Mackie E. J., Masuno H., Dwyer N. K., Olivecrona T., and Scow R. O. 1989 Lipoprotein lipase in myocytes and capillary endothelium of heart: immunocytochemical study. Am. J. Physiol. 256: E818–E828. 2735404 10.1152/ajpendo.1989.256.6.E818

