# Supplementary material for: Regulation of plasma lipid homeostasis by hepatic lipoprotein lipase in adult mice
Source: J Lipid Res. 2016 Jul;57(7):1155–61. doi: 10.1194/jlr.M065011 (PMC4918845; doi:10.1194/jlr.M065011)
Supplement: Supplemental Data [file supp_57_7_1155__index.html]

Regulation of Plasma Lipid Homeostasis by Hepatic Lipoprotein Lipase in Adult Mice — Regulation of plasma lipid homeostasis by hepatic lipoprotein lipase in adult mice — Supplemental Data 

# Regulation of plasma lipid homeostasis by hepatic lipoprotein lipase in adult mice

## Supplemental Data

- Supplemental Figures (.pdf, 298 KB) - Supplemental Fig.S1-S4
