# Supplementary material for: Regulation of plasma lipid homeostasis by hepatic lipoprotein lipase in adult mice
Source: J Lipid Res. 2016 Jul;57(7):1155–61. doi: 10.1194/jlr.M065011 (PMC4918845; doi:10.1194/jlr.M065011)
Supplement: Supplemental Data [file 10.1194_M065011_jlr.M065011-1.pdf]

Supplementary Data:

Regulation of Plasma Lipid Homeostasis by Hepatic Lipoprotein Lipase in Adult Mice

Gan Liu[1,2*], Jun-Nan Xu[1*], Dong Liu[3,1*], Qingli Ding[1], Meng-Na Liu[3,1], Rong Chen[2,4], Mengdi Fan[1,5], Ye Zhang[1,2], Chao Zheng[5], Da-Jin Zou[2,4], Jianxin Lyu[3], Weiping J. Zhang[1,2]

[1]Department of Pathophysiology, [2]Obesity and Diabetes Research Center, Second Military Medical University, Shanghai 200433, China

[3]The Key Laboratory of Laboratory Medicine, Ministry of Education of China, Wenchou Medical University School of Laboratory Medicine and Life Sciences, Wenchou, Zhejiang 325035, China

[4]Department of Endocrinology, Changhai Hospital, Shanghai 200433, China

[5]Department of Endocrinology, the Second Affiliated Hospital, Wenchou Medical University, Wenchou, Zhejiang 325000, China

* They contributed equally to the work.

Supplementary information:

- Methods
- Figures S1-S4

Materials and Methods

LPL activity assay. Briefly, 10 μl samples were mixed with 16 μl anhydrous emulsion (6% triolein, 0.36% lecithin, 0.01% ^{3}H-Triolein in glycerol), 74 μl BSA heparin mixture (3% BSA, 0.005‰ heparin sodium, 0.2% NaCl, 0.3M Tris-HCl, pH 8.5), 10 μl heat-inactivated fast rat serum and 90 μl PBS, and incubated at 37℃ for 60 min. Then, 3.25 ml extraction solvent (21% methanol, 44% chloroform, 35% heptane) and 1.05 ml potassium carbonate-borate buffer (0.7% potassium carbonate, 0.3% borate, pH10.5) were added to each tube, and the tubes were vortexed again and centrifuged at 3000g for 15 min. One ml of the upper aqueous phase were removed, mixed with 4 ml of scintillator liquid, and counted in a spectrometer (Beckman LS 6500). To abolish LPL activity, samples were incubated with 1M NaCl, and LPL activities were obtained by total enzyme activities subtracted NaCl-treated enzyme activities.

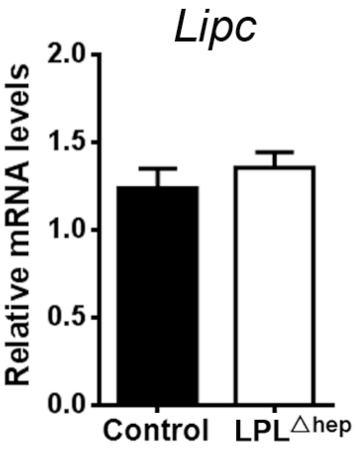

Supplementary Fig.S1. Deletion of hepatic *Lpl* did not affect the expression levels of *Lipc* mRNA in the liver. *Lipc* mRNA levels were detected by quantitative RT-PCR, and normalized by 36B4 as internal control. n=5 per group. P>0.05 vs control.

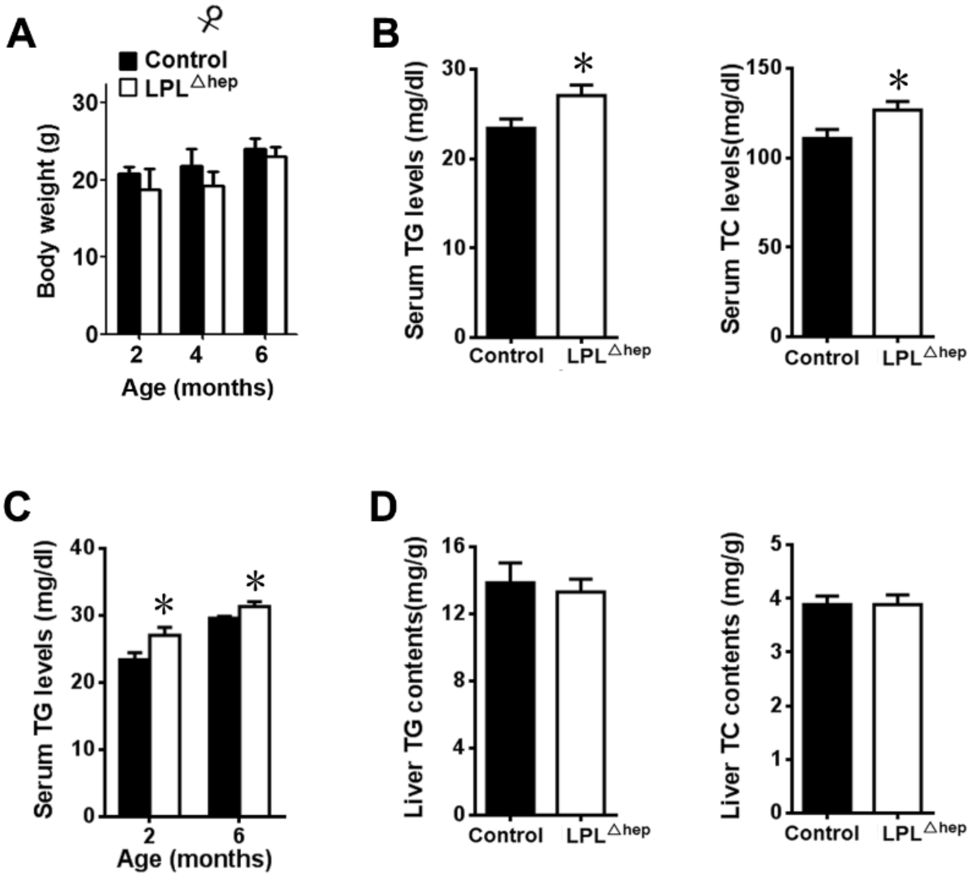

Supplementary Fig.S2. Disruption of liver LPL altered plasma lipid metabolism in female mice. The control and *Lpl*$^{\triangle hep}$ female mice at the age of 2~6 months were fed normal chow diet. (**A**) Body weight was not affected at the age of 2~6 months. (**B**) Fasting plasma TG and TC levels at the age of 2 months. (**C**) Fasting plasma TG levels at the indicated ages. (**D**) The TG and TC contents in the liver were not affected at 2 months of age by the deletion of hepatic *Lpl*. The mice were fasted for 6h. Results represent the mean ± SEM. *, $p<0.05$ vs. control, n=6-8 for each group.

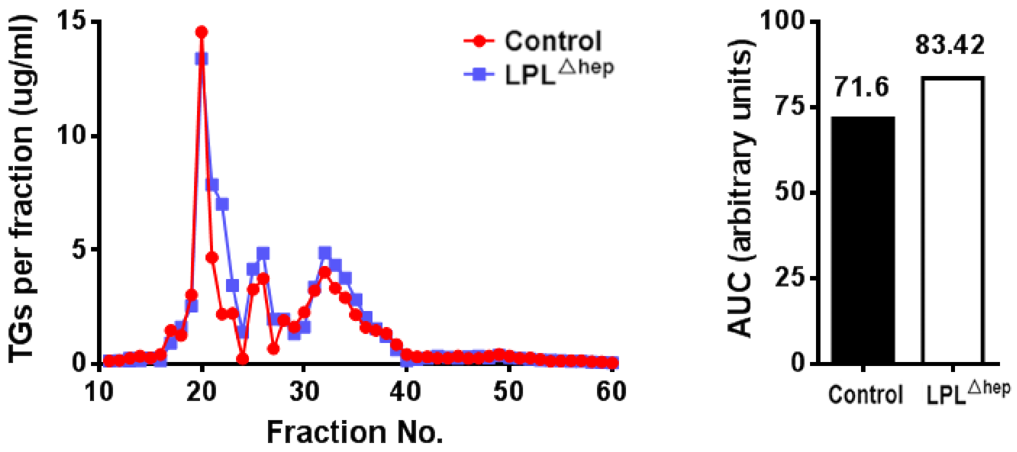

Supplementary Fig. S3. TG profile in plasma lipoprotein. Plasma samples pooled from 5 mice of each group were resolved by Superose 6 column, and the eluates collected in 0.5 ml by fractions were determined for TG contents. Area under curve (AUC) was plotted with the indicated values.

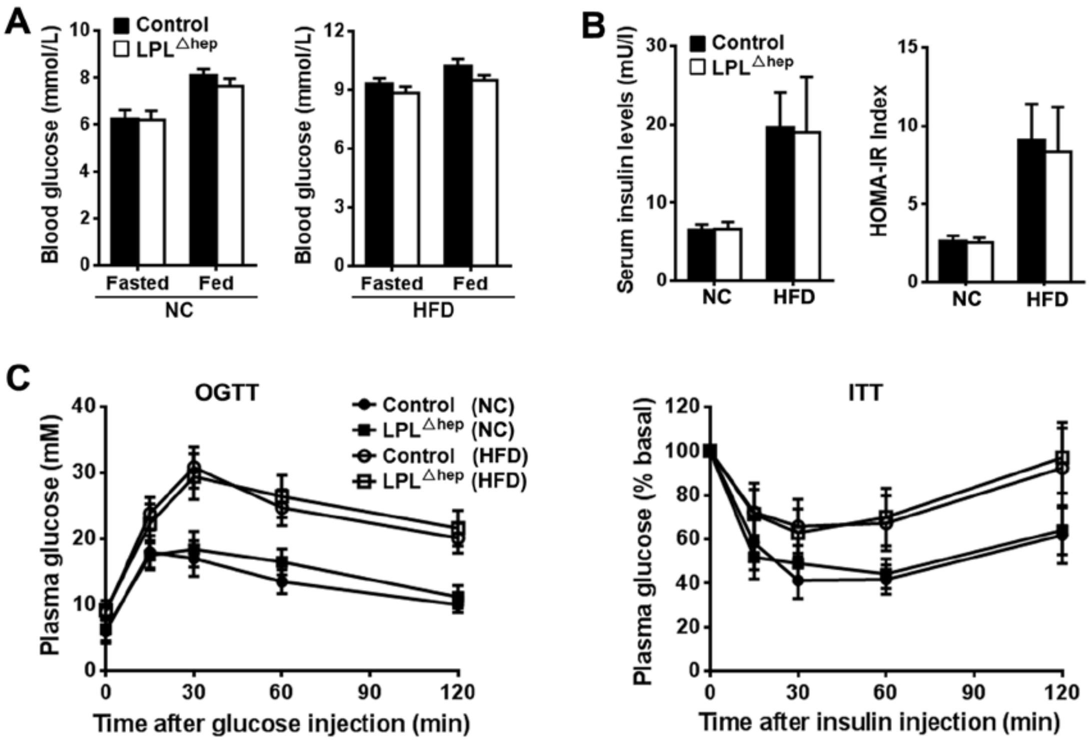

Supplementary Fig.S4. Deletion of liver LPL did not affect glucose homeostasis. The male control and mutant mice were fed the normal chow (NC) or high-fat diet (HFD) for 3 months. (**A**) Blood glucose levels under fasted or fed condition. (**B**) Fasting plasma insulin levels and HOMA-IR index of chow-fed mice. (**C**) Oral glucose tolerance test and insulin tolerance test showed that deletion of hepatic Lpl did not alter glucose metabolism of the mice on the normal chow or high-fat diet. The mice were administered with glucose (2g/kg body weight) by gavage or i.p. injected with 0.75U/kg of insulin, and their blood glucose levels were determined at the indicated time points. Results represent the mean ± SEM. n=4-6 each.
